# A roadmap for heart-liver co-management in MASLD

**DOI:** 10.1016/j.mmr.2026.100061

**Published:** 2026-08-12

**Authors:** Xiao-Dong Zhou, Nicholas W S Chew, Ming-Hua Zheng

**Affiliations:** MAFLD Research Center, Department of Hepatology, the First Affiliated Hospital of Wenzhou Medical University, Wenzhou 325000, Zhejiang, China; Key Laboratory of Diagnosis and Treatment for the Development of Chronic Liver Disease in Zhejiang Province, Wenzhou 325000, Zhejiang, China; Department of Cardiology, National University Heart Centre, National University Health System, Singapore 119074, Singapore; Yong Loo Lin School of Medicine, National University of Singapore, Singapore 119228, Singapore; MAFLD Research Center, Department of Hepatology, the First Affiliated Hospital of Wenzhou Medical University, Wenzhou 325000, Zhejiang, China; Key Laboratory of Diagnosis and Treatment for the Development of Chronic Liver Disease in Zhejiang Province, Wenzhou 325000, Zhejiang, China

**Keywords:** Metabolic dysfunction-associated steatotic liver disease (MASLD), Heart-liver crosstalk, Cardiovascular-kidney-liver-metabolic (CKLM) syndrome, Multiorgan medicine, Integrated care

Metabolic dysfunction-associated steatotic liver disease (MASLD) represents a major component of cardiometabolic disease, challenging traditional organ-based approaches to chronic disease management [1]. Increasing recognition of bidirectional liver-cardiovascular interactions through shared metabolic, inflammatory, and vascular pathways has provided the biological rationale for developing heart-liver co-management frameworks [2]. In 2025, major advances in disease classification and disease-modifying therapies marked a turning point in MASLD, transforming it from a condition with limited therapeutic options into a disease with emerging targeted treatment [3]. In 2026, the field is moving beyond therapeutic advances and recognition of cardiometabolic-liver interactions toward the development of integrated frameworks, longitudinal heart-liver evidence platforms, and emerging approaches to multiorgan clinical trial design that will advance heart-liver co-management (Fig. 1). However, major challenges remain in establishing longitudinal evidence frameworks, defining optimal therapeutic strategies, and translating this concept into a sustainable model of cardiometabolic medicine.

**Fig. 1:**
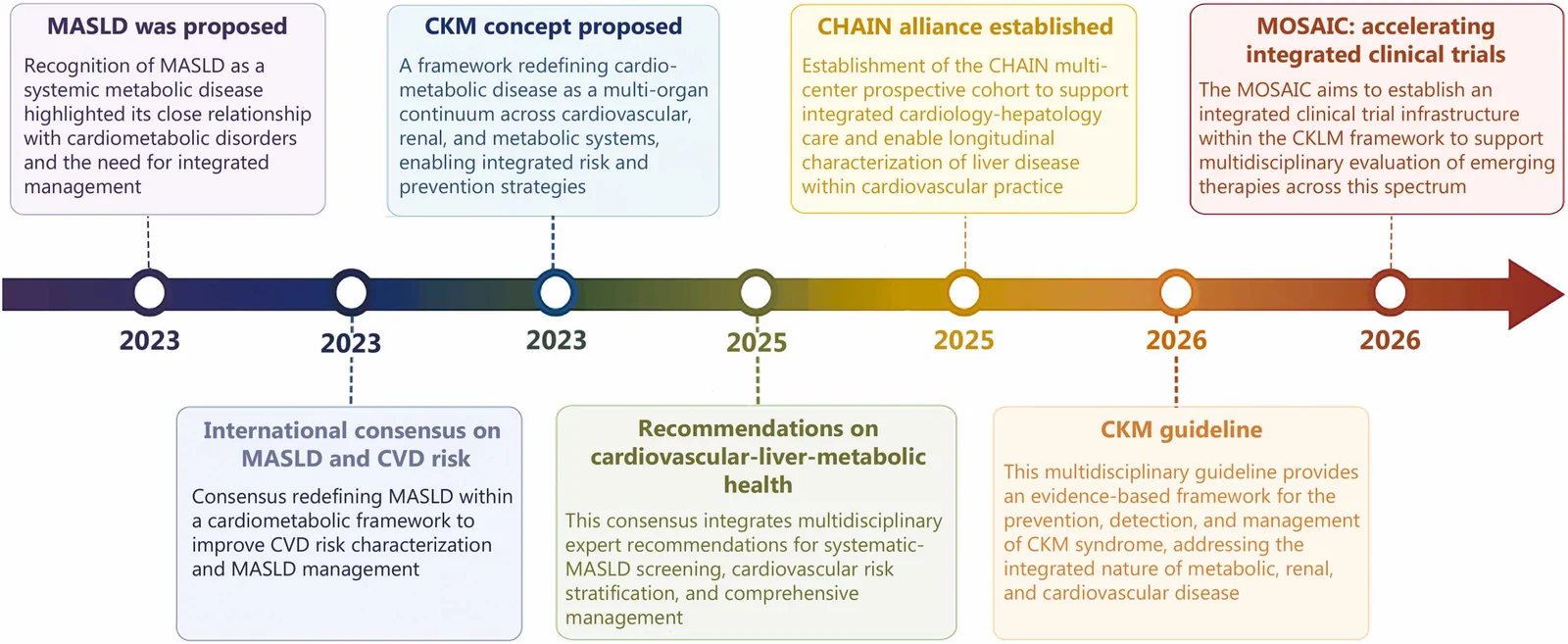
Evolution toward integrated heart-liver-metabolic care in MASLD. This figure illustrates the evolution of MASLD management from liver-focused disease recognition toward integrated heart-liver-metabolic care. Key transitions include the redefinition of fatty liver disease as MASLD, the emergence of disease-modifying therapies, the integration of MASLD into cardiovascular risk frameworks, the expansion of CKM toward CKLM concepts, and the establishment of prospective evidence and clinical implementation platforms, including the CHAIN cohort, CHAIN alliance, and MOSAIC clinical trial initiative. These recent developments mark a shift from conceptual recognition to evidence generation and system-level implementation. MASLD. Metabolic dysfunction-associated steatotic liver disease; CKM. Cardiovascular-kidney-metabolic; CKLM. Cardiovascular-kidney-liver-metabolic; CHAIN. CardiHepatic Alliance for Integrated Nexus; MOSAIC. Metabolic multiOrgan Science Accelerating Innovation in Clinical Trials; CVD. Cardiovascular disease.

A unified conceptual framework is a prerequisite for advancing heart-liver co-management. The cardiovascular-kidney-metabolic (CKM) framework has fundamentally reshaped the understanding of cardiometabolic disease by recognizing the interconnected nature of metabolic, cardiovascular, and renal dysfunction, and is increasingly being incorporated into research agendas, clinical guidelines, and care pathways [4]. However, the liver remains incompletely integrated within this paradigm despite its central role in systemic metabolic homeostasis and its close bidirectional relationship with cardiovascular disease (CVD). Growing recognition of this gap has led to increasing calls for extending CKM toward a cardiovascular-kidney-liver-metabolic (CKLM) framework [5]. Establishing a unified CKLM framework would provide a foundation for future mechanistic studies, longitudinal risk assessment, therapeutic innovation, and multidisciplinary care models [6].

Establishing a unified conceptual framework must be accompanied by a corresponding evolution in evidence generation. Current knowledge of heart-liver interactions remains largely derived from cross-sectional human studies and reductionist preclinical models, limiting the ability to resolve temporal causality and bidirectional inter-organ communication. Future research should therefore adopt integrated mechanistic and clinical approaches. These should combine longitudinal multi-omics, inter-organ experimental platforms, and animal models that better recapitulate dynamic heart-liver metabolic interactions. Equally important is the development of prospective longitudinal clinical cohorts incorporating repeated liver phenotyping within cardiovascular populations to define disease trajectories in real-world practice. Integration of multimodal data through artificial intelligence and machine learning may further facilitate dynamic risk prediction, patient phenotyping, and individualized therapeutic strategies across the heart-liver axis. In this context, the CardiHepatic Alliance for Integrated Nexus (CHAIN) cohort represents a prospective evidence platform for heart-liver co-management, integrating serial vibration-controlled transient elastography into cardiovascular care to generate longitudinal data on liver-CVD trajectories [7].

This evolving paradigm calls for a shift in therapeutic strategies [8]. Rather than targeting individual organs, emerging therapies, including glucagon-like peptide-1 receptor agonists, dual incretin receptor agonists, and sodium-glucose cotransporter 2 inhibitors, should increasingly be viewed as modulators of systemic cardiometabolic dysfunction, with potential roles in combination strategies to achieve broader effects across the liver, heart, vasculature, and adipose tissues [9]. Future therapeutic development should therefore focus on identifying cardiometabolic phenotypes most likely to benefit from these network-level interventions, rather than treating hepatic or CVD in isolation. This evolution also calls for a redesign of clinical trial frameworks [10]. Beyond traditional organ-specific endpoints, future studies should incorporate integrated phenotyping, longitudinal assessment across multiple organ systems, and composite outcomes that better capture coordinated cardiometabolic responses. Emerging initiatives such as Metabolic multiOrgan Science Accelerating Innovation in Clinical Trials (MOSAIC) aim to promote multiorgan approaches to clinical trial design by integrating shared mechanisms, patient phenotyping, and cross-organ outcomes. Adaptive or platform trial designs may further provide opportunities to evaluate combination strategies within a unified heart-liver therapeutic framework.

The next challenge is building integrated care systems that can support heart-liver co-management at scale. Future implementation of heart-liver co-management will require coordinated multidisciplinary care supported by integrated risk assessment, longitudinal data sharing, and harmonized clinical pathways. The development of CKM clinical practice guidelines provides an important precedent for translating multisystem disease concepts into clinical practice and may inform future efforts to establish evidence-based guidance for heart-liver co-management [4]. Cardiologists and hepatologists should jointly lead cardiovascular prevention and liver disease management, while endocrinologists and primary care physicians coordinate metabolic optimization, early identification, and long-term follow-up. Equally important, integrated evidence platforms capable of linking longitudinal phenotyping across specialties will be needed to align research with clinical decision-making. Such implementation frameworks may ultimately enable heart-liver co-management to become a practical component of routine cardiometabolic care.

As advances in disease biology, evidence generation, therapeutic innovation, and clinical implementation continue to converge, heart-liver co-management may provide a foundation for the next generation of cardiometabolic medicine.

## Abbreviations

CKLM: Cardiovascular-kidney-liver-metabolic
CKM: Cardiovascular-kidney-metabolic
CVD: Cardiovascular disease
MASLD: Metabolic dysfunction-associated steatotic liver disease

## CHAIN Consortium

Hong-Lian Bai, Yan-Zhen Bi, Jin Chai, En-Qiang Chen, Jian-Dong Chen, Jun-Kang Chen, Li-Zhen Chen, Baizhen Deqing, Jie Deng, Liang Deng, Jian-Long Dong, Ren Cheng-Han Fan, Cheng-Cheng Feng, Xiao-Qin Gao, Yan-Hang Gao, Yu-Feng Gao, Ning Geng, Man Gu, Hao Guan, Ting-Yu Guo, Chun-Yi He, Cheng-Lv Hong, Fang-Jie Hou, Yue-Yan Hu, Yan Huang, Yi-Jia Huang, Yu-Li Huang, Yue Huang, Ze-Bing Huang, Fan-Pu Ji, Hai-Bing Jiang, Ya-Kun Jiang, Shu-Jun Jiang, Xing-Xing Jin, Kebinuer Tuerxun, Bo Li, Hai-Ming Li, Jia Li, Jun Li, Jun-Feng Li, Min Li, Xian-Lun Li, Xiao-Ping Li, Yan Li, Yi-Ling Li, Yi-Wen Li, You-Fu He, Hong-Xia Liang, Xiao Liang, Su Lin, Chang Liu, Feng-Hua Liu, Jing Liu, Li-Gai Liu, Yan-Fei Liu, Ying-Wu Liu, Yue Liu, Cheng-Zhi Lu, Zheng-Jie Lu, Qiang Luo, Xin-Hua Luo, Yue Luo, Jing-Bo Lv, Ke-Ke Lv, Li Ma, Li-Min Ma, Zhen-Qi Ma, Yi-Long Man, Xiao-Fan Miao, Si-Jia Ning, Hong Peng, Ping Peng, Xiao-Ping Peng, Xiang-Yuan Pu, Bao-Xin Qian, Xi-Yun Rao, Ming Ren, Yuan-Yuan Ren, Shali Shalaimaiti, Cong-Xiang Shao, De-Liang Shen, Jing-Yu Shen, Ming-Wei Sheng, Yu Shi, Yao-Hong Song, Guan-Yue Su, Yun-Juan Su, Jing Sun, Wei Sun, Xin-Yi Sun, Wei-Yan Tai, Jia-Ke Tang, Qian Tong, Liu-Hua Wang, Dong-Fei Wang, Feng-Mei Wang, Ming-Wei Wang, Qi Wang, Qian Wang, Xiao-Qi Wang, Ya-Dong Wang, Xiao-Ping Wu, Ming-Feng Xia, Rui Xiang, Yong-Ning Xin, Fan-Qi Xu, Hui-Juan Xu, Wei-Feng Xu, Yuan-Ying Xu, Ming-Hui Xu, Hong-Ju Yang, Hui Yang, Li-Xia Yang, Song Yang, Zhi-Luo Yang, Hua Ye, Jun-Zhao Ye, Qing Ye, Zhong Ye, Hong-Shan Yin, Ge Yu, Song-Yuan Yu, Xia Yu, Abulaiti Yusufujiang, Cheng-Long Zhang, Hao-Tian Zhang, Hong-Yan Zhang, Li-Ting Zhang, Shuai-Ran Zhang, Xiao-Qian Zhang, Xin-Gang Zhang, Yan-Liang Zhang, Yan Zhang, Yin-Qiang Zhang, Jing-Jing Zhao, Wei Zhao, Wen-Ting Zhao, Yang Zhao, Ming-Hua Zheng, Yu-Bao Zheng, Bi-Hui Zhong, Jing-Yuan Zhou, Jing Zhou, Ruo-Qi Zhou, Xi-Qiao Zhou, Xiao-Dong Zhou, Xue-Qian Zhou, and Meng-Meng Zhu.

## Ethics approval and consent to participate

Not applicable.

## Funding

This work was supported by the Noncommunicable Chronic Diseases-National Science and Technology Major Project (2026ZD0557000 to MHZ), the National Key R&D Program of China (2023YFA1800801), and the National Natural Science Foundation of China (82070588, 82370577).

## CRediT authorship contribution statement

XDZ is conceptualization and writing of the original draft. NWSC is responsible for review and editing. MHZ is responsible for conceptualization, supervision, writing, and editing. All authors read and approved the final manuscript.

## Competing interests

The author declares that they have no competing interests.

## Data Availability

Not applicable.
